# Knowledge, attitude, and practice regarding atrial fibrillation among neurologists in central China: A cross‐sectional study

**DOI:** 10.1002/clc.23361

**Published:** 2020-03-24

**Authors:** Jing Shen, Yuanpeng Xia, Shiyi Cao, Zuxun Lu, Quanwei He, Man Li, Hong Wang, Ying Bi, Shaoli Chen, Bo Hu, Fei Cao

**Affiliations:** ^1^ Department of Neurology, Union Hospital, Tongji Medical College Huazhong University of Science and Technology Wuhan China; ^2^ School of Public Health, Tongji Medical College Huazhong University of Science and Technology Wuhan China; ^3^ Department of Neurology, The First Affiliated Hospital, School of Medicine Shihezi University Shihezi China

**Keywords:** atrial fibrillation, attitude, knowledge, neurologists, practice

## Abstract

**Background:**

Physicians' knowledge and practice of atrial fibrillation (AF) are determinants of the efficacy of thromboprophylaxis.

**Hypothesis:**

This study aimed to investigate physicians' knowledge, attitude, and practice toward AF, to analyze the influencing factors, and to provide data to support departments that develop health policies.

**Methods:**

A cross‐sectional study was carried out from October 1, 2016, to March 31, 2018. A standard‐structured anonymous questionnaire was completed by each participant through face‐to‐face interviews.

**Results:**

A total of 611 doctors from 38 hospitals were responded to this survey. The mean of the total score of the questionnaire was 21.59 ± 3.559 (total score of the questionnaire was 36), and the mean scores of knowledge, attitude, and practice were 6.86 ± 1.70, 6.13 ± 1.35, and 8.59 ± 2.21, respectively. The doctor' s knowledge, practice scores, and total scores were positively correlated with the education level and the workplace. The influencing factors that affect doctors' knowledge, attitudes, and practice scores including education level, professional title, working years, hospital grade, and hospital location.

**Conclusions:**

There was still a big gap in neurologists' knowledge and practice about AF. It is necessary to strengthen the continuous improvement of doctor training to improve the management of AF.

## 1 INTRODUCTION

Ischemic stroke is the disease with the highest incidence and mortality in China.^1^ Based on Chinese ischemic stroke subclassification (CISS) classification,^2^ one‐third of ischemic strokes are cardiogenic strokes, of which the primary cause is atrial fibrillation (AF). During AF, the blood clot could fall off and flow with the blood, leading to embolism of multiple organs, especially the brain.^3^ Previous studies have shown that from 2001 to 2012, the prevalence of AF increased 20‐fold.^4^ The Framingham Heart Study indicated that AF could raise the risk of ischemic stroke by more than 5‐fold, compared with subjects without AF.^5^ Therefore, stroke prevention is the principal management priority in patients with AF, which is determined by both the physician and the patient.

Although AF augments the risk of stroke, there are still defects in preventing stroke caused by AF. Physicians play an essential role in the management of AF thus physicians may be well placed to provide counseling and education to patients on all aspects of anticoagulation, including self‐management. However, a lack of knowledge often leads to suboptimal communication and decision‐making. Clinicians' cognition, attitudes, and behavior related to stroke caused by AF may determine patients' prognosis and medication compliance.^6^ There have been few investigations focusing on clinicians in terms of AF‐related stroke, and research in this area has not been reported in China. Here, we conducted a cross‐sectional survey among neurologists in Hubei Province in Central China at different hospital levels. We sought to evaluate their knowledge, attitude, and practice toward AF and to elucidate the influencing factors. According to the influencing factors, we could find the corresponding method to improve the relevant defects from the doctor's point of view.

## 2 METHODS

### 2.1 Ethics statement and consent to participate

The study was conducted strictly following the Declaration of Helsinki and was approved by the Research Ethics Committee of Tongji Medical College, Huazhong University of Science and Technology, Wuhan, China. All respondents were aware of the purpose of the investigation and signed a written informed consent form.

### 2.2 Settings and participators

A cross‐sectional study was carried out in Hubei province, which is located in central China. Hubei province has 13 major cities, including the provincial capital city of Wuhan. According to the principle of random cluster sampling, we randomly selected 42 hospitals as the investigation settings by the geographical and four of them declined to be included in the study. Finally, 38 hospitals were eventually enrolled for the study, including all levels of hospitals (level III, level II, level І). The hospital levels were classified by the standards set by the national health commission of China. Based on the evaluation levels and location, hospitals were divided into grade A tertiary hospital and other grade hospitals or in Wuhan and outside of Wuhan. We conducted face‐to‐face interviews with clinicians. The inclusion criteria for participating physicians were as follows: (a) Working in a public hospital, all of the participators were neurologists; and (b) Had a physician certificate and legal medical qualifications. Seven hundred and ten doctors answered questions from the questionnaire, 99 doctors refused to answer fully, and the valid questionnaires were 611.

### 2.3 Data collection

Each participant completed a standard‐structured anonymous questionnaire. Investigators had received training given by the Stroke Quality Control Center of Hubei Province and Hubei Preventive Medicine Association. Then, the physicians got a face‐to‐face interview. Experts designed the questionnaire items, and 30 doctors were given it in advance to ensure its clarity and feasibility. The final questionnaire was composed of three parts: demographic characteristics (part I), issues about AF assessment and screening (part II), and issues about the preventive medication (part III). In part II and part III, there were several questions about knowledge, attitudes, and practice. The AF‐related knowledge scale involved 12 questions, each question was awarded a point if the physician selected the correct answer, and a zero score (0 points) was given if the answer was incorrect. We used the same method to calculate the attitude and practice score. The AF‐related attitudes scale involved five questions and the practice scale involved seven questions. The full score of a physician's knowledge, attitudes, and practice around AF were 12 points, 8 points, and 16 points, respectively, and the total possible score of the questionnaire was 36 points.

### 2.4 Statistical analysis

Data from the completed questionnaires were double entered into the EpiData software package (version 3.1) and expressed as mean ± SD for continuous data, and as counts and percentages for categorical data. The distribution of categorical data was examined by the chi‐square test. Continuous data were compared using Student's *t*‐test or the analysis of variance (ANOVA). Logistic regression analysis was performed to assess the risk factors associated with knowledge, attitude, and practice score. The average score of knowledge, attitude, and practice was used as the critical values to group the knowledge, attitude, and practice score, respectively. Then the grouped variables were used as the dependent variables to further multivariate analysis. The trending chi‐square test is used to explore the trend relationship between variables. A two‐sided *P* < .05 was considered statistically significant. The analyses were performed using Statistical Product and Service Solutions (SPSS) software version 22.0 (IBM Corporation, Armonk, New York).

## 3 RESULTS

### 3.1 Sociodemographic characteristics of the participants and score of each part of the questionnaire

Table 1 presents the sociodemographic characteristics of the participants and the score of each part of the questionnaire. In total, 611 doctors, including 345 males (56.5%) and 266 females (43.5%), were participated in the survey. The average age was 36.08 ± 8.41 years old. Among the participants surveyed, the mean of the total score was 21.59 ± 3.56, and the mean scores of knowledge, attitude, and practice were 6.86 ± 1.70, 6.13 ± 1.35, and 8.59 ± 2.21, respectively. Each part scored less than 60% of the corresponding total score. By comparative analysis, there were significant differences in knowledge score, attitude score, practice score, and total score. The doctors' knowledge score was positively correlated with age, education attainment, professional title, hospital level, and the workplace. Doctors older than 40 years old have a higher knowledge score than doctors under 40. The higher the level of education, the higher the professional title, the higher the knowledge score. Doctors who worked in grade A tertiary hospitals have a higher score than doctors working in other grade hospitals. Doctors working in Wuhan hospitals scored higher than doctors working outside Wuhan hospitals. In terms of attitude scores, only the difference between the professional title and the place of work was statistically significant. The deputy chief physician, attending physician, and resident physician scored higher than the chief physician and interns. Physicians working in Wuhan scored higher than doctors working outside of Wuhan. In terms of practice score, the differences between different levels of education, professional titles, working years, and hospital grades were statistically significant. The higher the level of education, the higher the professional title, the longer the working years, the higher the practice score. The doctors working in the grade A tertiary hospitals score higher than other grade hospitals.

**TABLE 1:** The characteristics of participants and score of each part of the questionnaire

Characteristic/items	n	%	Knowledge score	Attitude score	Practice score	Total score
Gender						
Female	266	43.5	6.82 ± 1.68	6.20 ± 1.38	8.59 ± 2.21	21.60 ± 3.61
Male	345	56.5	6.90 ± 1.72	6.08 ± 1.32	8.60 ± 2.20	21.58 ± 3.52
*t*			0.597	−1.075	0.059	−0.085
*P*			.551	.283	.953	.932
Age (years old)						
<40	435	71.2	6.77 ± 1.73	6.15 ± 1.34	8.50 ± 2.18	21.42 ± 3.49
>=40	176	28.8	7.09 ± 1.59	6.09 ± 1.37	8.82 ± 2.26	22.00 ± 3.70
*t*			−2.12	0.552	−1.652	−1.825
*P*			.034	.581	.099	.068
Education attainment						
Below the university degree	9	1.5	6.11 ± 1.05	6.22 ± 1.39	7(7,8)	19.44 ± 1.81
Bachelor's degree	246	40.3	6.41 ± 1.73	6.03 ± 1.40	8(7,10)	20.83 ± 3.67
Graduate degree	289	47.3	7.12 ± 1.64	6.20 ± 1.30	9(7,10)	22.01 ± 3.42
PhD	67	10.9	7.51 ± 1.49	6.19 ± 1.36	9(8,10)	22.84 ± 3.26
*F*			12.329	0.758	12.038	9.346
*P*			.001	.518	.007	.001
Professional title						
Intern	35	5.7	5.63 ± 1.65	5.60 ± 1.32	7.89 ± 2.26	19.11 ± 3.56
Resident doctor	169	27.7	6.93 ± 1.69	6.18 ± 1.35	8.07 ± 1.96	21.18 ± 3.34
Attending physician	220	36	6.86 ± 1.71	6.19 ± 1.30	8.84 ± 2.26	21.89 ± 3.44
Associate chief physician	132	21.6	6.96 ± 1.61	6.24 ± 1.33	8.89 ± 2.28	22.10 ± 3.60
Archiater	55	9	7.18 ± 1.66	5.84 ± 1.48	8.96 ± 2.17	21.98 ± 3.92
*F*			5.447	2.424	5.131	6.211
*P*			.001	.047	.001	.001
Duration of working in the neurology clinical (years)						
0–5	219	35.8	6.74 ± 1.70	6.02 ± 1.36	8.14 ± 2.08	20.89 ± 3.42
6–10	157	25.7	6.84 ± 1.63	6.18 ± 1.29	8.85 ± 2.26	21.87 ± 3.42
11–20	160	26.2	6.91 ± 1.79	6.29 ± 1.33	8.86 ± 2.23	22.06 ± 3.67
20–	75	12.3	7.17 ± 1.63	6.01 ± 1.45	8.83 ± 2.21	22.01 ± 3.77
*F*			1.304	1.569	4.953	4.52
*P*			.272	.196	.002	.004
Hospital level						
Grade A tertiary hospital	437	71.5	6.98 ± 1.64	6.12 ± 1.34	8.76 ± 2.21	21.86 ± 3.56
Other grade hospital	174	28.5	6.57 ± 1.81	6.16 ± 1.37	8.17 ± 2.14	20.90 ± 3.43
*t*			2.656	−0.262	3.032	3.144
*P*			.008	.794	.003	.002
Work place						
In Wuhan	253	41.4	7.35 ± 1.69	6.39 ± 1.35	8.72 ± 2.27	22.46 ± 3.77
Outside Wuhan	358	58.6	6.52 ± 1.62	5.95 ± 1.32	8.50 ± 2.15	20.97 ± 3.27
*t*			6.11	4.042	1.234	5.091
*P*			.001	.001	.218	.001
Total	611	100	6.86 ± 1.70	6.13 ± 1.35	8.59 ± 2.21	21.59 ± 3.56

### 3.2 The distribution and ratio for each item

Table 2 showed the distribution and answering ratio for each item. In each part, the item of high accuracy and consistency was different. In terms of knowledge, some item with a higher correct rate but a particular gap and others with a very low correct rate and the item that needs to be greatly improved. In terms of attitudes, doctors showed a high degree of consistency, and the response rate of each question exceeded 90%, indicated the doctors' willingness to prevent and manage AF. In terms of practice, the subject with different response rates. The results reflected the lack and deficiency of doctors in some aspects.

**TABLE 2:** Answers to each item in each part of the questionnaire

Items	n	%
Knowledge(up to 12 points)		
Does atrial fibrillation increase the risk of stroke?	604	98.9
How much risk of stroke is increased in atrial fibrillation?	312	51.1
Does atrial fibrillation increase the risk of stroke recurrence?	605	99.0
Is paroxysmal atrial fibrillation and persistent atrial fibrillation the same risk of stroke?	110	18.0
Proportion of atrial fibrillation in patients with ischemic stroke	102	16.7
The best treatment for stroke patients with atrial fibrillation	488	79.9
Does paroxysmal atrial fibrillation require anticoagulant therapy?	475	77.7
Does new atrial fibrillation require anticoagulant therapy?	485	79.4
Did you know and used the CHADS_2_ score?	239	39.1
Did you know and used the CHADS_2_‐VAS_C_ score?	180	29.5
Did you know and used the HAS‐BLED score?	122	20.0
What do you think is the reasonable range of INR for patients who take oral warfarin?	471	77.1
Attitude(up to 8 points)		
Do you think it is necessary to screen for atrial fibrillation in patients with unexplained ischemic stroke?	609	99.7
Do you think it is necessary to promote a wearable ECG monitor?	558	91.3
Do you have the need to learn atrial fibrillation knowledge?	575	94.1
For patients with long‐term oral warfarin, what frequency do you think is appropriate to monitor INR?	554	90.7
What measures can you take to improve the INR monitoring rate?(A total of 4 options, 1 point for each selection)	/	/
Practice(up to 16 points)		
Have you had atrial fibrillation screening for patients with unexplained stroke?	521	85.3
Which techniques have you used to screen your patients for atrial fibrillation? (1 point for each selection)	/	/
What ECG monitors are there in your hospital? (1 point for each selection)	/	/
Has your hospital‐organized training for atrial fibrillation screening in stroke patients?	332	54.3
Has your hospital‐organized anticoagulant therapy training?	415	67.9
Do you recommend that patients with long‐term oral anticoagulants regularly monitor their INR values?	597	97.7
Is your hospital equipped with equipment to monitor INR?	364	59.6

Abbreviations: INR, International Normalized Ratio; ECG, electrocardiogram.

### 3.3 Associated factors

A multivariate logistic regression analysis was performed to assess the risk factors associated with knowledge, attitude, and practice score. Table 3 displayed the influencing factors of each group. The analysis found that for knowledge score, those who had Bachelor's degree (*P* = .002) got a lower score, but those who worked in Wuhan hospitals (*P* = .001) were more likely to get higher scores. For attitude score, those who worked for 11 to 20 years and worked in the grade A tertiary hospitals (*P* = .001) got higher scores. For practice score, those who had below the university degree (*P* = .048) got a lower score but those who worked in the grade A tertiary hospitals (*P* = .026) got higher scores.

**TABLE 3:** Factors associated with knowledge, attitude, and practice score

Variables	Knowledge score				Attitude score				Practice score			
			95% CI				95% CI				95% CI	
	*P*	OR	Lower	Upper	*P*	OR	Lower	Upper	*P*	OR	Lower	Upper
Gender	.599	0.917	0.664	1.266	.576	1.098	0.791	1.524	.712	0.941	0.684	1.296
Age (years)	.233	1.242	0.87	1.773	.958	0.99	0.692	1.416	.813	0.933	0.522	1.665
Education attainment (Ref. = PhD)												
Below the university degree	.295	0.462	0.109	1.963	.884	1.116	0.256	4.87	.048	0.11	0.012	0.981
Bachelor's degree	.002	0.385	0.208	0.711	.511	0.829	0.473	1.452	.561	0.826	0.434	1.573
Graduate degree	.457	0.794	0.433	1.458	.515	0.832	0.479	1.446	.441	0.795	0.443	1.426
Professional title (Ref. = Archiater)												
Intern	.002	0.229	0.091	0.571	.180	0.556	0.235	1.312	.463	0.613	0.166	2.264
Resident doctor	.665	0.87	0.463	1.633	.282	1.402	0.758	2.595	.251	0.522	0.172	1.584
Attending physician	.274	0.711	0.386	1.310	.325	1.349	0.743	2.45	.48	0.711	0.276	1.832
Associate chief physician	.442	0.776	0.405	1.484	.289	1.142	0.746	2.67	.698	0.856	0.39	1.878
Duration of working in the neurology clinical (Ref. = 0–5)												
6–10	.146	0.669	0.39	1.15	.38	1.206	0.794	1.831	.445	1.257	0.699	2.262
11–20	.216	0.699	0.397	1.232	.032	1.591	1.04	2.435	.467	1.287	0.652	2.539
20–	.301	0.742	0.421	1.306	.769	0.924	0.545	1.565	.502	1.389	0.532	3.627
Hospital level	.064	0.717	0.504	1.02	.988	0.997	0.696	1.428	.026	0.613	0.399	0.943
Work place	.001	0.452	0.318	0.641	.001	0.543	0.388	0.761	.376	0.864	0.626	1.193

Abbreviation: CI, confidence interval.

Based on the results of the multivariate analysis, we conducted a further trend analysis (Table 4). The results showed that for the knowledge score, the score level had a precise positively correlation with the level of education (*P* = .001; *R* = 0.206). However, the trend between knowledge score and professional title and working years was pointless. For practice score, the scores correlated with the level of education (*P* = .01; *R* = 0.105), professional title (*P* = .001; *R* = −0.143), and the length of working years (*P* = .002; *R* = 0.127). Although the correlation coefficient was small, it was statistically significant. For the attitude score, no similar trend was found.

**TABLE 4:** The results of trend analysis

Variables	Group of knowledge score		Group of attitude score		Group of practice score	
	<7 points, n (%)	≥7 points, n (%)	<6 points, n (%)	≥6 points, n (%)	<9 points, n (%)	≥9 points, n (%)
Education attainment						
Below the university degree	5 (1.5)	4 (1.2)	3 (1.2)	6 (1.6)	8 (2.7)	1 (0.3)
Bachelor's degree	137 (51.5)	109 (31.6)	99 (40.9)	147 (39.8)	126 (42.3)	120 (38.3)
Graduate degree	106 (39.8)	183 (53.0)	116 (47.9)	173 (46.9)	140 (47.0)	149 (47.6)
PhD	18 (6.8)	49 (14.2)	24 (9.9)	43 (11.7)	24 (8.1)	43 (13.7)
*χ* ^2^	26.593		0.129		6.724	
*P*	.001		.719		.01	
Person's *R*	0.209				0.105	
*P*	.001				.04	
Professional title						
Intern	25 (9.4)	10 (2.9)	21 (8.7)	14 (3.8)	19 (6.4)	16 (5.1)
Resident doctor	67 (25.2)	102 (29.6)	63 (26.0)	106 (28.7)	100 (33.6)	69 (22.0)
Attending physician	98 (36.8)	122 (35.4)	84 (34.7)	136 (36.9)	105 (35.2)	115 (36.7)
Associate chief physician	56 (21.1)	76 (22.0)	49 (20.2)	83 (22.5)	54 (18.1)	78 (24.9)
Archiater	20 (7.5)	35 (10.1)	25 (10.3)	30 (88.1)	20 (6.7)	35 (11.2)
*χ* ^2^	3.057		0.326		12.495	
*P*	.08		.568		.001	
Person's *R*					−0.143	
*P*					.04	
Duration of working in the neurology clinical						
0–5	100 (37.6)	119 (34.5)	95 (39.3)	124 (33.6)	125 (41.9)	94 (30.0)
6–10	70 (26.3)	87 (25.2)	61 (25.2)	96 (26.0)	74 (24.8)	83 (26.5)
11–20	69 (25.9)	91 (26.4)	52 (21.5)	108 (29.3)	69 (23.2)	91 (29.1)
20–	27 (10.2)	48 (13.9)	34 (14.0)	41 (11.1)	30 (10.1)	45 (14.4)
*χ* ^2^	1.685		0.765		9.793	
*P*	.194		.382		.002	
Person's *R*					0.127	
*P*					.04	

## 4 DISCUSSION

The present study explored neurologists' knowledge, attitude, and practice of AF. First, it showed that doctors working in neurology in Central China have insufficient knowledge and implementation of AF. Second, doctors had a high level of awareness about the risk of AF, and the need and methods of treatment, but there was insufficient understanding of further subdivision issues, such as the understanding of AF classification and the assessment of pretreatment related methods. Third, factors affecting doctors' knowledge, attitudes, and practice scores, including education level, professional title, working years, hospital grade, and hospital location. Some of these intervention factors may serve as a breakthrough to improve doctors' knowledge, attitude, and practice.

Some studies about AF knowledge have been conducted focusing on patients and found that there is a poor understanding of stroke and AF.^7^ However, few studies are centering on doctors. In a survey of European physicians treating AF patients, they found that major gaps existed in physicians' knowledge and skills across all domains of AF care.^8^ Clinicians are needed to master the correct assessment and reasonable treatment of stroke patients with AF; however, the result is not ideal.^9^ The significant findings of this study showed that, in knowledge score, attitude score, practice score, and total score, there were significant differences. There were a difference in the age, level of education, professional title, working lifetime, working hospital level and location. Also, the professional title, working hospital level, location (in or outside Wuhan), and working lifetime affected the four scores. The reasons may be as follows: The older the doctor, the longer the working time, the more work experience, the more opportunities he has received the appropriate training. In China, grade A tertiary hospitals have better resources and more opportunities to acquire the latest knowledge. The overall level of doctors' education is higher in grade A tertiary hospitals, especially in Wuhan. As a provincial capital city, Wuhan has distinct geographical advantages, convenient transportation, more quality resources, and learning opportunities for doctors to learn.

From Table 2, it can be observed that doctors have certain characteristics about the knowledge about AF. For the problem of high popularity and wide publicity, the awareness rate is high, but for further subdivision problems, such as the classification and evaluation methods of the AF, the awareness rate is far below our expected. As a medical professional, doctors are responsible for informing patients about the risk, outcome, and appropriate treatment of the disease. However, in this survey, the correct rate of these problems is not satisfactory. The difference between the adverse effects of paroxysmal AF and persistent AF is a long‐standing phenomenon. The Loire Valley Atrial Fibrillation Project with a large real‐world cohort did not show a difference between patients with paroxysmal AF and permanent AF after multivariate adjustments.^10^ However, recent analyses demonstrated that paroxysmal AF was associated with a higher risk of stroke.^11^ Our study found that only 18.2% (103/611) of doctors knew that the risk of stroke in paroxysmal AF was less. The rate of ischemic stroke among patients with nonvalvular AF averages 5%/year.^12^ However, only 16.7% (103/611) of doctors knew the correct proportion. The results suggested Chinese doctors have deeply insufficient basic knowledge about AF‐related strokes, which may lead to inappropriate counseling and education for the patients. We need to increase investment in workforce and material resources and provide more relevant lectures.

Major risk factors associated with AF have been used to develop risk prediction models and anticoagulant drug guidance for embolic stroke, such as the CHADS_2_, the CHADS_2_‐VASc and the HAS‐BLED scoring systems.^13^, ^14^, ^15^ However, in this study, we found that the application of the CHADS_2,_ the CHADS_2_‐VASc, and the HAS‐BLED scoring system was not high, and the total rate of knowledge and use was only 39.1%, 29.5%, and 20.0%, respectively. The result is frustrating because if a doctor does not understand the score scales, he cannot judge the risk of embolism, the advantage, and disadvantage of anticoagulant drugs. Therefore, promoting the awareness of the CHADS_2_, CHADS_2_‐VASc, and HAS‐BLED scores and increasing the use of them are basic needs.

The influencing factors that affect doctors' knowledge, attitudes, and practice scores included education level, professional title, working years, hospital grade, and hospital location. Some of them are intervenable factors, while others are nonintervention factors. Intervention factors include: doctor's education level, professional title, workplace, and nonintervention factors include: working years, hospital level. However, there is a certain degree of connection between the intervening factors and the nonintervention factors. For example, doctors can gain more knowledge about AF by strengthening self‐learning. They can also improve their knowledge by participating in hospital‐organized training. In their continuous learning, they can improve their cultural level and promote themselves to the higher professional title. From the hospital's point of view, the grade A tertiary hospitals should hold more professional knowledge training to improve the awareness of doctors' knowledge about AF, thereby improving the level of attitude and practice of doctors with AF, and choosing a reasonable solution for the secondary prevention and long‐term management. These methods are also applicable to hospitals outside Wuhan.

## 5 CONCLUSIONS

Collectively, we all believe that the lack of knowledge about AF in patients has led to the lack of management and anticoagulant therapy for AF, but did not realize that the doctor's knowledge of AF is not enough. The results of our study demonstrate that the neurologists surveyed in central China had inadequate knowledge and practice on AF. There is scope for improvement for neurologists in central China concerning AF knowledge and practice. Hospital administrators and government health officials should conduct targeted training to improve the awareness of AF‐related knowledge among physicians at all levels to improve the prevention and treatment of AF and AF‐related stroke.

### 5.1 Study limitations

Unavoidably, this study has some limitations that need to be acknowledged. First, given the limitations of the cross‐sectional design, we are unable to assess and describe the effects of interventions on improving the doctor's knowledge, attitude, and practice. However, the findings can be valuable for providing directed improvement measures and interventions for AF. Second, in this study, some indicators involved are too small, such as the degree of education. Third, this study used a face‐to‐face interview format. Because the number of hospitals involved is large and the staffing is insufficient, the whole research involves a long time and has a certain impact on the timeliness of the research. In the future, network interviews should be used to improve efficiency.

## CONFLICT OF INTEREST

The authors declare no potential conflict of interests.
